# Mg^2+^ Supplementation Mitigates Metabolic Deficits Associated With TRPM7 Disruption

**DOI:** 10.1002/jcp.70042

**Published:** 2025-04-25

**Authors:** Severin Boulassel, Pascale C. F. Schreier, Anna M. Melyshi, Johanna Berger, Peter S. Reinach, Katharina Jacob, Ingrid Boekhoff, Andreas Breit, Timo D. Müller, Susanna Zierler, Thomas Gudermann, Noushafarin Khajavi

**Affiliations:** ^1^ Walther Straub Institute of Pharmacology and Toxicology, LMU Munich Munich Germany; ^2^ Ophthalmology Department Wenzhou Medical University Wenzhou People's Republic of China; ^3^ Institute of Diabetes and Obesity Helmholtz Center Munich Munich Germany; ^4^ German Center for Diabetes Research (DZD) Düsseldorf Germany; ^5^ Institute of Pharmacology, Medical Faculty Johannes Kepler University Linz Linz Austria; ^6^ German Center for Lung Research Munich Germany

**Keywords:** glucose metabolism, Mg2+ supplementation, pancreatic β‐cell, proliferation, TRPM7

## Abstract

Transient receptor potential channel subfamily M member 7 (TRPM7) regulates cellular and systemic Mg^2+^ homeostasis through its channel domain and induces protein phosphorylation via its kinase domain. We recently found that mice with selective deletion of *Trpm7* in β‐cells develop glucose intolerance and declines in insulin secretion, primarily due to the impaired enzymatic activity of this protein. Accumulating evidence suggests that Mg^2+^ supplementation effectively mitigates the detrimental effects of TRPM7 disruption in various cell types. However, the impact of Mg^2+^ supplementation on metabolic impairments caused by TRPM7 inactivation remains unclear. In the present study, we found that Mg^2+^ supplementation significantly ameliorates glucose intolerance observed in high‐fat‐fed TRPM7 kinase‐deficient mice (*Trpm7* ^*R/R*^). However, our ex vivo analysis of islets isolated from *Trpm7* ^*R/R*^ mice revealed that Mg^2+^ supplementation does not enhance glucose‐induced insulin secretion. Instead, the improvement appears to be partially driven by enhanced insulin sensitivity and increased β‐cell proliferation. The pharmacological analysis in MIN6 cells showed that inhibiting TRPM7 with either NS8593 or VER155008 disrupts β‐cell proliferation. These effects mimicked the phenotype seen in *Trpm7* ^*R/R*^ mice. We attribute this impairment to diminished ERK1/2 signaling, which suppressed PDX1 expression, while Mg^2+^ supplementation in vitro partially restored ERK1/2 phosphorylation levels. Collectively, Mg^2+^ supplementation enhances glucose metabolism in *Trpm7* ^*R/R*^ mice and mitigates the ERK1/2 signaling disruptions and proliferation arrest induced by TRPM7 inactivation in vitro. These findings provide compelling evidence that Mg^2+^ supplementation can reverse the adverse metabolic and cellular phenotypes associated with the loss of TRPM7 function.

## 1 Introduction

Magnesium (Mg^2+^) is the fourth most abundant mineral in the human body and serves as a crucial cofactor for numerous enzymes involved in metabolic processes. It plays an essential regulatory role in glucose metabolism, facilitating glucose transport across cell membranes, and is vital for hepatic gluconeogenesis (Alghobashy et al. 2018; Mittermeier et al. 2019; Morais et al. 2017; Elderawi et al. 2018). Additionally, it has been suggested that Mg^2+^ contributes to the regulation of insulin secretion in pancreatic β‐cells and phosphorylation of insulin receptors in target cells (Günther 2010; Kostov 2019).

Transient receptor potential cation channel subfamily M member 7 (TRPM7) is a bifunctional protein that combines a divalent cation‐selective channel with an atypical α‐type serine‐threonine kinase domain (Nadler et al. 2001; Runnels et al. 2001). Its channel moiety is a key regulator of cellular and whole body Mg^2+^ homeostasis in mammals (Ryazanova et al. 2010). The kinase moiety of TRPM7 has been implicated in the regulation of various cellular processes, such as proliferation, cell growth, migration, apoptosis, differentiation, and exocytosis (Zierler et al. 2017). Early studies suggested that TRPM7 kinase activity is crucial for the channel function (Runnels et al. 2001; Schmitz et al. 2003). While it was initially proposed that the kinase domain is essential for channel gating (Runnels et al. 2001), later research revealed that the kinase domain primarily modulates TRPM7 channel sensitivity to inhibition by Mg·nucleotides. The Mg·nucleotide inhibition plays a critical role in modulating TRPM7 channel activity in response to fluctuations in cellular energy levels, ensuring that ion transport is tightly coupled to the metabolic state of the cell (Demeuse et al. 2006; Nadler et al. 2001; Schmitz et al. 2003). The nucleotide‐dependent regulation is mediated by a specific binding site within the kinase domain, and the inhibition is abolished in phosphotransferase activity‐deficient point mutant of TRPM7 (Demeuse et al. 2006).

Further studies demonstrated that inactivation of TRPM7 kinase also impacts the TRPM7 protein stability and cellular dynamics. Cells expressing kinase‐deficient TRPM7 exhibited faster protein degradation, increased ubiquitination, and greater intracellular retention of the channel compared to cells expressing WT protein. Notably, HEK‐293T cells transiently expressing the K1646R mutant displayed significantly lower protein expression levels than those expressing the WT protein (Cai et al. 2018). These findings highlight the critical role of the TRPM7 kinase domain in regulating channel function, stability, and trafficking.

Recently, our research along with those of others highlighted the pivotal role of TRPM7 in preserving β‐cell function under both physiological and metabolically stressed conditions (Altman et al. 2021; Khajavi, Beck, et al. 2023). Through studies using β–cell–specific *Trpm7*‐knockout mice (*βTrpm7*‐KO), we demonstrated that the loss of TRPM7 function results in progressive β‐cell dysfunction and impaired insulin secretion. We attributed this diminished insulinotropic response in β*Trpm7*‐KO mice to transcriptional alterations in β‐cells, resulting in decreased expression of essential genes involved in insulin biosynthesis and cell cycle regulation, that are likely due to the loss of TRPM7 enzymatic activity. Additionally, we found that impaired TRPM7 kinase activity contributes to the gradual loss of both β‐cell identity and diminished β‐cell proliferation (Khajavi, Beck, et al. 2023).

Multiple studies have demonstrated that Mg^2+^ supplementation can effectively reverse the impaired phenotypes associated with TRPM7 disruption (Gupta et al. 2023; Ryazanova et al. 2010; Yee et al. 2011). In embryonic stem cells, inactivation of TRPM7 kinase resulted in inhibition of cellular proliferation, a condition that can be rescued by Mg^2+^ supplementation (Ryazanova et al. 2010). In *Trpm7*‐deficient embryos, Mg^2+^ supplementation alleviated developmental arrest by reversing the overexpression of oxidative stress‐related genes and enhancing mitochondrial function. Additionally, Mg^2+^ supplementation restored the expression of transcription factors essential for cell proliferation in these embryos, highlighting the crucial role in mitigating the effects of loss of TRPM7 function (Gupta et al. 2023). In zebrafish, supplementary Mg^2+^ partially rescued the exocrine pancreatic defects in *trpm7* mutants by enhancing cell cycle progression and cell growth (Yee et al. 2011).

The current study evaluates the ability of supplementary Mg^2+^ to reverse the impaired metabolic phenotype induced by TRPM7 disruption. Our findings indicate that Mg^2+^ supplementation enhances glycemic control and β‐cell proliferation in *Trpm7* ^*R/R*^ mice. Notably, we showed that the beneficial effect of Mg^2+^ on glycemic control operates independently of TRPM7 kinase activity in the machinery of insulin secretion. Through pharmacological analyses, we identified TRPM7 as a pivotal regulator of key signaling pathways involved in β‐cell proliferation. Our in vitro experiments revealed that Mg^2+^ supplementation effectively counteracts the impaired signaling pathways caused by TRPM7 inhibition in β‐cells.

## 2 Materials and Methods

### 2.1 Mouse Strains and Genotyping Procedures

*Trpm7tm1.1Mkma C56BL/6* (K1646R, *Trpm7* ^*R/R*^) mice were provided by Masayuki Matsushita (Okayama University Medical School, Okayama, Japan). The *Trpm7* ^*R/R*^ mice were generated by homologous recombination in embryonic stem cells, replacing the WT *Trpm7* gene with a point mutant *Trpm7* allele encoding the TRPM7 K1646R protein. The K1646 mutation, which substitutes a conserved lysine with arginine, leads to diminished kinase activity while preserving the overall integrity of the channel domain (Kaitsuka et al. 2014). Mice were backcrossed to C57BL/6 ( ≥ 6 generations). Mice were housed in ventilated cages at the animal facility of the Walther Straub Institute of Pharmacology and Toxicology, LMU Munich, Munich, Germany. Heterozygous K1646R animals were bred to obtain age‐ and sex‐matched homozygous WT and homozygous *Trpm7* ^*R/R*^ mice. For genotyping, DNA was extracted from ear fragments using the Mouse Direct PCR Kit (Biotool). DNA samples were analyzed by PCR using a set of allele‐specific oligonucleotides (Metabion). Sequence information is previously described. Genotyping of *Trpm7* ^*R/R*^ mice was performed as previously described (Mittermeier et al. 2019). Male and female mice were fed an obesogenic diet (Research Diets, D12331), containing 58% kcal from fat and 0.22% of Mg^2+^, beginning at 10−12 weeks of age for 8 weeks. After this period, mice received D12331 with or without 0.75% supplemental Mg^2+^. Mice were single‐ or group‐housed on a 12‐h light/12‐h dark cycle at 22°C with free access to food and water. Mice were maintained under these conditions for a maximum of 16 weeks.

### 2.2 Characterization of Glucose Homeostasis

For the determination of glucose tolerance, mice (male and female) were fasted overnight (16 h). Basal blood glucose was sampled, and glucose was administered as an intraperitoneal (i.p.) injection at a dose of 2 g/kg body weight (20% w/v d‐glucose from Sigma‐Aldrich in dPBS from Gibco). Blood samples were obtained from the tail vein. Blood glucose levels were measured by glucometer (Contour Next, Ascensia Diabetes care) before (0 min) and at 15, 30, 60, and 120 min after injection. For investigation of blood parameters, blood was collected after euthanasia using lithium heparin micro sample tubes (Sarstedt), immediately cooled on ice, centrifuged at 2000×*g* and 4°C for 10 min, and plasma stored at −80°C. Plasma insulin was quantified by an insulin ELISA kit (ALPCO).

### 2.3 Islet Isolation and Determination of Insulin Secretion

Islets were isolated from 26‐week‐old male and female mice. Isolation of pancreatic islets was performed as previously described (Khajavi et al. 2018). In brief, the pancreas was perfused by injection of 3 mM Collagenase‐P (Roche) (0.4 mg/mL) in HBSS containing 25 mM HEPES and 0.5% (w/v) BSA into the common bile duct. Isolated islets were recovered for 48 h in RPMI 1640 (Thermo Fisher Scientific) in humidified 5% CO_2_, at 37°C. After this period, islets were used for functional assessments. Before determination of insulin secretion, islets were equilibrated for 1 h in KRB buffer (115 mM NaCl, 4.5 mM KCl, 1.2 mM KH_2_PO_4_, 2.6 mM CaCl_2_, 1 mM MgCl_2_, 10 mM HEPES, 20 mM NaHCO_3_, 0.2% w/v BSA, pH 7.4) with 2.8 mM glucose. Determination of insulin secretion from the islets was performed in 12‐well plates containing 600 μL KRB (8 islets/well, five independent experiments performed in triplicate). After 1 h preincubation in KRB with 2.8 mM glucose, islets were incubated for 1 h in 20 mM glucose or 300 μM tolbutamide. Released insulin was measured in the supernatant using an insulin ELISA kit.

### 2.4 Western Blot Analysis

Western blot analysis was performed as previously described. A total of 20 μg of protein was loaded, resolved on 12.5% Tris‐HCl SDS‐PAGE, and blotted onto a PVDF membrane (Immobilon). Membranes were blocked for 1 h using 5% BSA or nonfat dried milk diluted in Tris‐buffered saline with 0.1% Tween 20 detergent at room temperature and incubated with primary antibodies (Table 1) at 4°C for 16 h. After washing, membranes were incubated with HRP‐conjugated secondary antibodies (Table 1) for 1 h at room temperature. Immunobound antibody was visualized with a chemiluminescent peroxidase substrate (Sigma‐Aldrich). ChemiDoc MP Imaging System (Bio‐Rad) was used for chemiluminescence detection. For the loading control, membranes were cut and incubated with an antibody against histone H3 for approximately 16 h at 4°C.

**Table 1:** Antibody details and specifications.

Antigen	Host species	Dilution	Source	Catalog number
Insulin	Guinea pig	Ready to use	Dako	IR002
Ki67	Rabbit	1:500	Abcam	Ab 15580
PDX1	Rabbit	1:500	Abcam	Ab134150
Histone H3	Rabbit	1:10000	Abcam	Ab1791
P‐ERK	Rabbit	1:1000	ThermoFisher	44‐680G
Tot. ERK	Mouse	1:1000	ThermoFisher	13‐6200
P‐AKT (S473)	Rabbit	1:1000	Cell Signaling	9271
Tot. AKT	Rabbit	1:1000	Cell Signaling	9272
Alexa Fluor 488 goat anti‐guinea pig	Goat	1:1000	ThermoFisher	A11073
Alexa Fluor 647 goat anti‐rabbit	Goat	1:1000	ThermoFisher	A21245
Goat Anti‐Rabbit IgG (H + L) HRP Conjugate	Goat	1:5000	Bio‐Rad	1,706,515
Goat Anti‐Mouse IgG (H + L) HRP Conjugate	Goat	1:5000	Bio‐Rad	1,706,516

### 2.5 Morphological Analysis

Standard hematoxylin and eosin staining on 10 μm cryosections of pancreas isolated from *Trpm7* ^*R/R*^ and control littermates was performed to assess the number of islets per pancreatic section. The size of β‐cells was measured by imaging randomly selected insulin‐positive cells at 400× and determined as the mean individual β‐cell cross‐sectional area for at least five islets per animal using ImageJ software. Investigators followed a blinded protocol during analysis.

### 2.6 Measurement of β‐Cell Proliferation

Pancreatic slices were prepared from *Trpm7* ^*R/R*^ and control littermates or MIN6 cells. To study β‐cell proliferation in pancreatic islets were co‐stained for insulin and Ki67. Antibodies and their working dilutions are listed in Table 1. Ki67‐insulin double‐positive cells were counted and divided by the total number of insulin‐positive cells per pancreatic section. To investigate cell proliferation in MIN6 cells, cells were preincubated for 24 h with 30 µM NS8593 or 10 µM VER155008. Cells were co‐stained then for insulin and Ki67. Ki67‐positive cells were counted and divided by the total cell number per slide.

### 2.7 Cell Culture

MIN6 cells were provided by Per‐Olof Berggren and Barbara Leibiger, Karolinska Institutet, Stockholm, Sweden. MIN6 cells were grown at 37°C and 5% CO_2_ in DMEM (Gibco) supplemented with 10% FBS (Gibco), 100 U/mL penicillin and 100 μg/mL streptomycin (Gibco), 1 mM sodium pyruvate (Gibco), 10 mM HEPES (Gibco), and 71 μM β‐mercaptothion (Gibco). Cells with approximately 60% confluence in 6 cm dishes were incubated with 30 µM NS8593 or 10 µM VER155008 for the western blot analysis or immunostaining. Cells were harvested 24 h after blocker exposure for western blot analysis. For Mg^2+^ supplementation, 10 mM MgCl_2_ was added to the culture medium. For immunostaining, cells were incubated for 24 h with the blockers.

### 2.8 Inductively Coupled Plasma Mass Spectrometry

The Mg^2+^ content was measured using inductively coupled plasma mass spectrometry (ICP‐MS) by ALS Scandinavia (Sweden). MIN6 cells were incubated overnight in DMEM medium with or without 10 mM MgCl_2_ in the presence of 30 µM NS8593 or DMSO (vehicle). Following incubation, cells were washed twice with dPBS and seeded with a density of five million cells per condition. The cell pellets were dried overnight at 70°C and stored at −80°C. Samples were then shipped on dry ice for ICP‐MS analysis.

### 2.9 Statistics

Data are expressed as mean ± SEM. *p* values less than 0.05 are considered significant. Graph presentations, curve fittings, statistics, and *p* values were obtained by Prism software (version 9.0.1; GraphPad). For comparison of two groups, *p* values were calculated by the unpaired two‐tailed Student's *t*‐test for parametric or the Mann−Whitney test for nonparametric distribution. For three or more groups, one‐way ANOVA with Bonferroni's multiple comparison were used for parametrically distributed data. Glucose tolerance tests were compared using two‐way ANOVA with Bonferroni's multiple comparison.

## 3 Results

### 3.1 Mg^2+^ Supplementation Improves Glucose Tolerance in TRPM7 Kinase‐Deficient Mice

**Figure 1:**
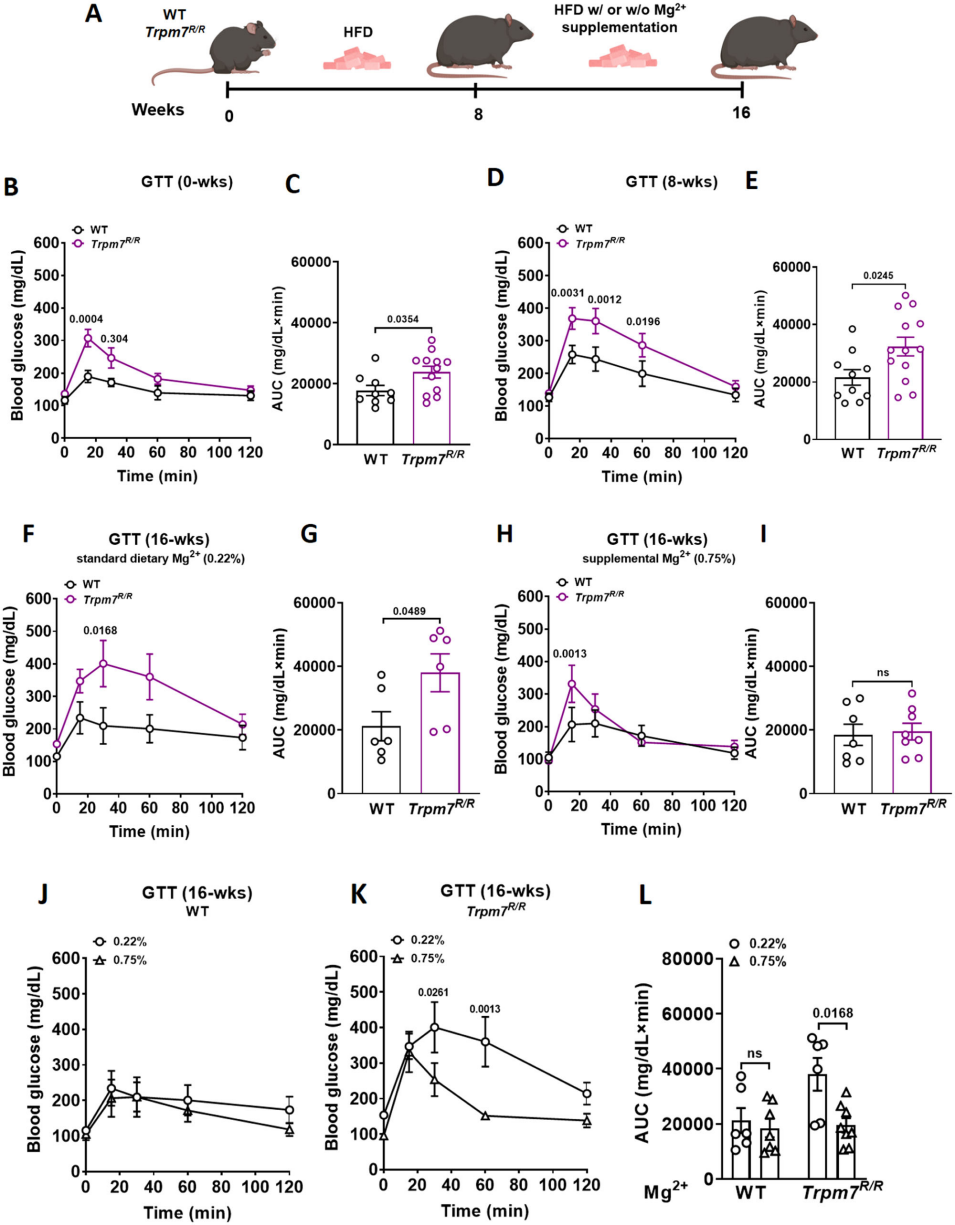
Mg^2+^ supplementation enhances glucose tolerance in *Trpm7* ^*R/R*^ mice. (A) Schematic of the feeding regimen for the mice. (B−L) Glucose tolerance tests (GTT) were performed after overnight fasting (*n* ≥ 6 mice per genotype). Blood glucose levels (mg/dL) were measured before and up to 2 h following an intraperitoneal (i.p.) injection of glucose (2 g/kg body weight) in wild‐type (WT) and *Trpm7* ^*R/R*^ mice. The panels show blood glucose levels (B, D, F, H, J, K) and the area under the curve (AUC in mg/dL × min; C, E, G, I, L) for mice aged 10−12 weeks (0‐weeks) (B, C), after 8 weeks on a high‐fat diet (HFD) with 0.22% Mg^2+^ (8‐weeks) (D, E), after 8 weeks on HFD with 0.22% Mg^2+^ concentration (16‐weeks) (F, G), and after 8 weeks on HFD with 0.75% Mg^2+^ supplementation (16‐weeks) (H, I). (J, K) GTT comparison between WT (J) and *Trpm7* ^*R/R*^ (K) under two different dietary Mg^2+^ concentrations. (L) Area under the curve (AUC in mg/dL × min) quantifying the GTT results from J and K. Data are presented as means ± SEM. Statistical significance was determined using two‐way ANOVA (B, D, F, H, J, K, L) or unpaired two‐tailed Student's *t*‐test (C, E, G, I). Individual values are represented by circles in the bar graphs. *p* values are indicated above the bars.

We compared the metabolic phenotype of a mouse model harboring a point mutation in the active site of the enzyme (*Trpm7* ^*R/R*^) with their control littermates on an 8‐week obesogenic diet (D12331) containing standard dietary Mg^2+^ (0.22%). Next, we evaluated the effects of 8 weeks of Mg^2+^ supplementation, provided at either standard (0.22%) or high (0.75%) concentration on this phenotype (Figure 1A). *Trpm7* ^*R/R*^ mice exhibited glucose intolerance as early as 10−12 weeks of age, which worsened following 8 weeks on an obesogenic diet (Figure 1B−E). Specifically, *Trpm7* ^*R/R*^ mice fed high‐fat diet (HFD) with 0.22% Mg^2+^ exhibited pronounced glucose intolerance compared to their control littermates (Figure 1F,G). Glucose tolerance was markedly improved in *Trpm7* ^*R/R*^ mice that received the 0.75% Mg^2+^ supplementation. No significant difference was observed in the area under the curve (AUC) between *Trpm7* ^*R/R*^ mice and their control littermates after 8 weeks on HFD supplemented with 0.75% Mg^2+^ (Figure 1H,I). Notably, *Trpm7* ^*R/R*^ mice showed a significant improvement in glucose tolerance after receiving 0.75% Mg^2+^ compared to *Trpm7* ^*R/R*^ mice on the standard dietary Mg^2+^ (0.22%) (Figure 1J). No differences in glucose tolerance were observed in WT mice under either of the two dietary Mg^2+^ conditions (Figure 1K,L).

### 3.2 Mg^2+^ Supplementation Does Not Improve Glucose‐Induced Insulin Secretion (GIIS) in Islets Isolated From TRPM7 Kinase‐Deficient Mice

**Figure 2:**
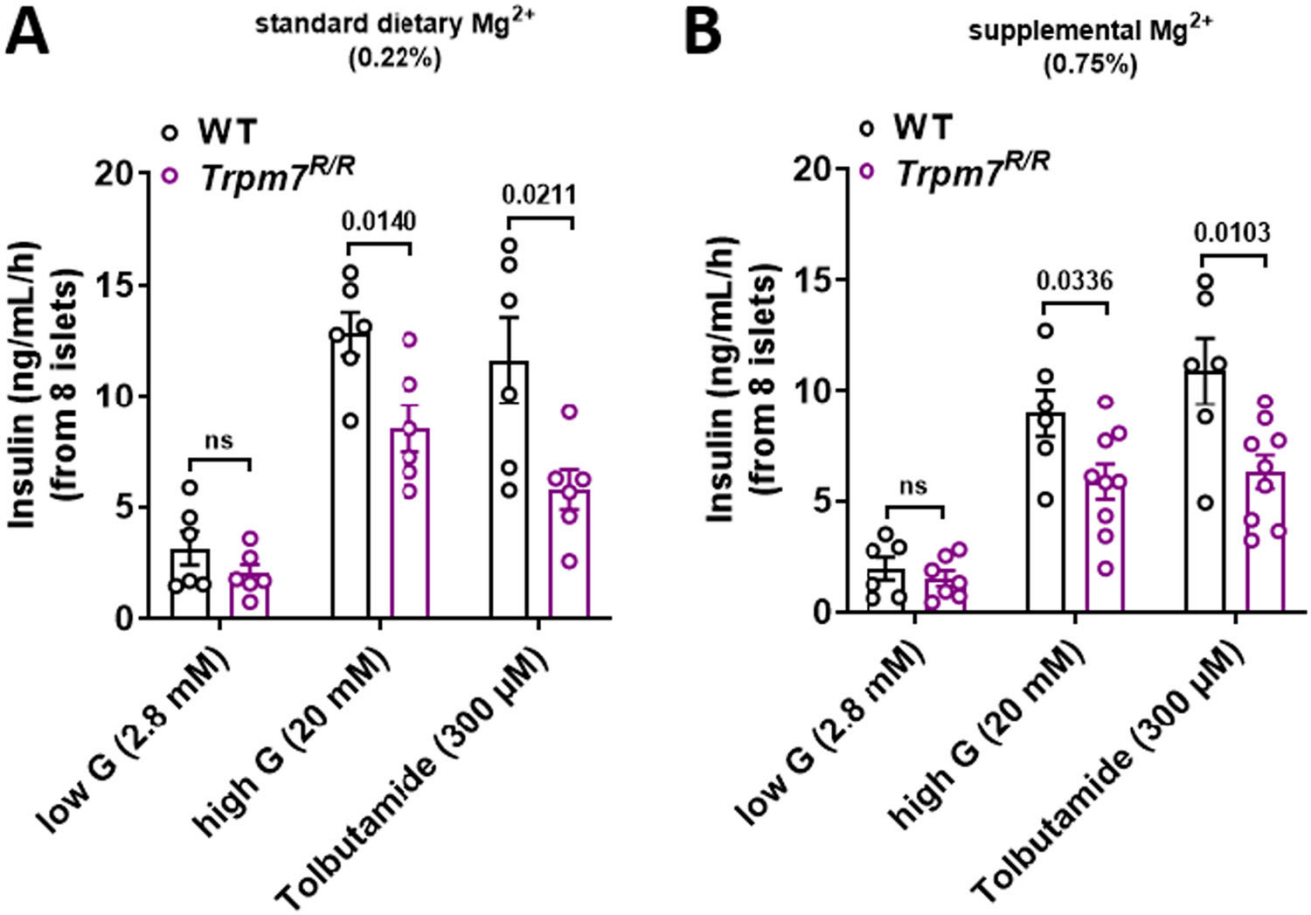
Mg^2+^ supplementation has no effect on GIIS in *Trpm7* ^*R/R*^ islets. (A, B) Insulin secretion (ng/mL/h/8 islets) was assessed in isolated islets from male and female *Trpm7* ^*R/R*^ and control littermate mice without (0.22% Mg^2+^) (A) and with Mg^2+^ supplementation (0.75% Mg^2+^) (B). Islets were incubated for 1 h with low glucose (2.8 mM), high glucose (20 mM), or 300 μM tolbutamide (*n* ≥ 5 mice per genotype, measured in duplicate). Released insulin was measured in the supernatant using an insulin ELISA kit. Data are presented as means ± SEM, and statistical significance was determined using one‐way ANOVA. Individual values are represented by circles in the bar graphs. *p* values are indicated above the bars.

Given the improved glycemic control in *Trpm7* ^*R/R*^ mice following 0.75% Mg^2+^ supplementation, we hypothesized that this enhancement may be attributed to an improvement in GIIS. In islets isolated from WT mice, increasing glucose concentration from 2.8 to 20 mM and inducing β‐cell membrane depolarization with 300 μM tolbutamide significantly enhanced insulin exocytosis, irrespective of whether mice had received the 0.22% or 0.75% Mg^2+^ diet. However, in islets isolated from *Trpm7* ^*R/R*^ mice, GIIS was significantly impaired on both diets when compared to control littermates. Furthermore, basal insulin secretion was lower in *Trpm7* ^*R/R*^ islets compared to WT controls. However, this difference did not reach statistical significance (Figure 2A,B).

### 3.3 Mg^2+^ Supplementation Reduces Fasting Blood Glucose and Improves Serum Insulin Levels in TRPM7 Kinase‐Deficient Mice

**Figure 3:**
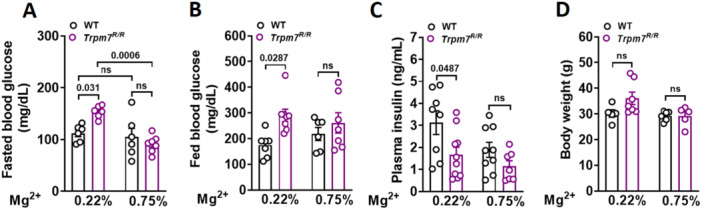
Mg^2+^ supplementation improves blood glucose and plasma levels of insulin in *Trpm7* ^*R/R*^ mice. (A, B) Blood glucose levels (mg/dL) were measured in *Trpm7* ^*R/R*^ and control littermate mice after 8 weeks of HFD and 8 weeks of varying doses of supplementary Mg^2+^, in fasted (A) and freely fed (B) states (*n* ≥ 6 per genotype). (C) Plasma insulin levels (ng/mL) were measured in freely fed *Trpm7* ^*R/R*^ and control littermate mice under the same conditions (*n* ≥ 6 mice per genotype). (D) Body weight after 8 weeks of HFD and 8 weeks of different dosages of supplementary Mg^2+^ (*n* ≥ 6 mice per genotype). Data are presented as means ± SEM, and statistical significance was determined using one‐way ANOVA. Individual values are represented by circles in the bar graphs. *p* values are indicated above the bars.

As we did not observe enhanced insulin secretion in *Trpm7* ^*R/R*^ mice following 0.75% Mg^2+^ supplementation, we hypothesized that the improved glucose tolerance might result from enhanced insulin sensitivity and glucose clearance. To explore this, we examined the metabolic phenotype of *Trpm7* ^*R/R*^ mice under two different dietary Mg^2+^ concentrations compared to their control littermates. After 8 weeks of supplementation with 0.75% Mg^2+^, *Trpm7* ^*R/R*^ mice displayed lower fasting blood glucose compared to *Trpm7* ^*R/R*^ mice on a standard Mg^2+^ diet. In contrast, fasting blood glucose levels in WT mice remained consistent regardless of dietary Mg^2+^ concentration (Figure 3A). Notably, with 0.75% Mg^2+^ supplementation, both fasting and fed blood glucose levels in *Trpm7* ^*R/R*^ mice more closely matched those of their control littermates (Figure 3A,B). Furthermore, *Trpm7* ^*R/R*^ mice on 0.75% Mg^2+^ diet exhibited plasma insulin levels similar to those of their control littermates (Figure 3C). No significant differences in body weight were observed between *Trpm7* ^*R/R*^ mice and control littermates following either 0.22% or 0.75% Mg^2+^ concentration (Figure 3D).

### 3.4 Mg^2+^ Supplementation Enhances β‐Cell Proliferation in TRPM7 Kinase‐Deficient Mice

**Figure 4:**
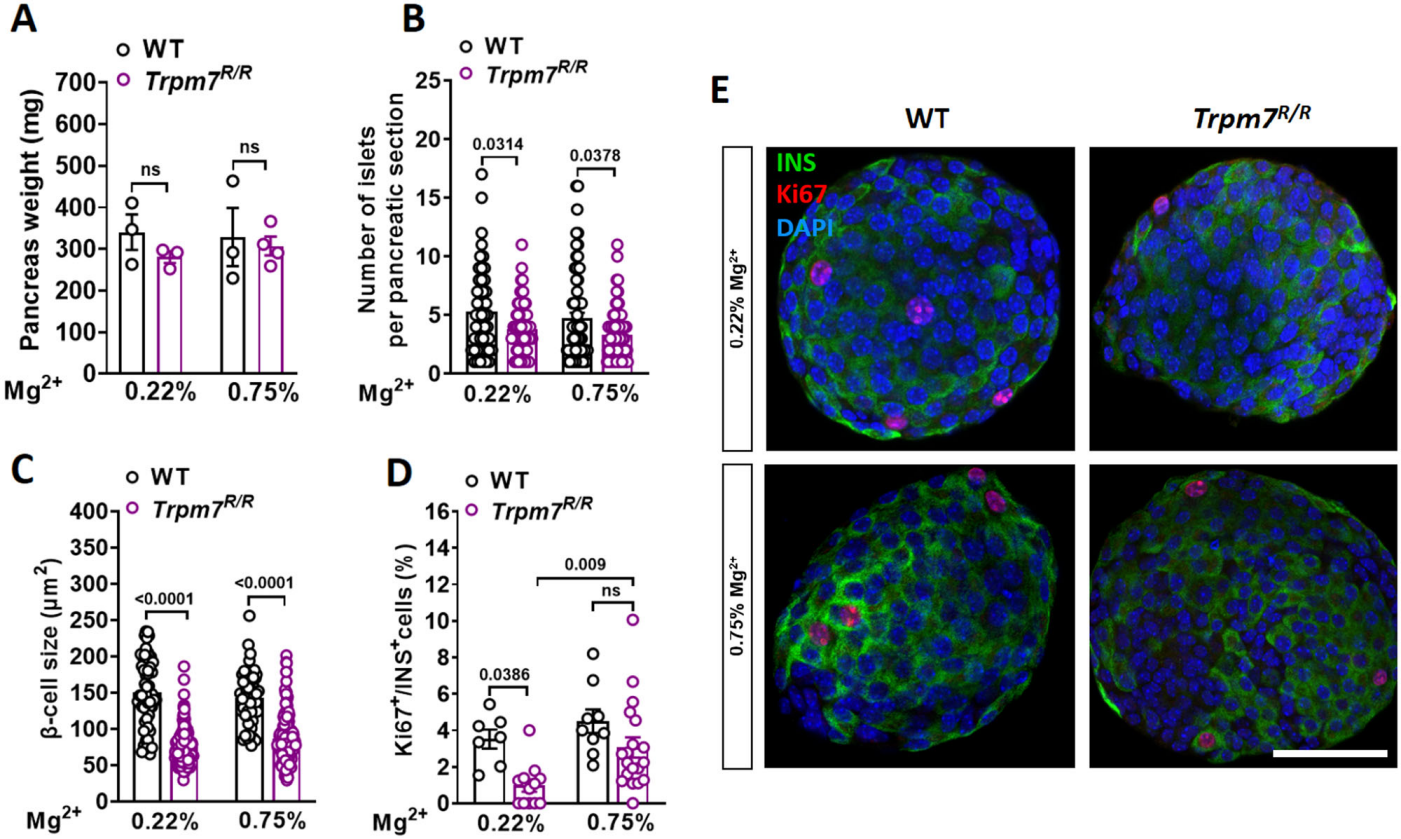
Mg^2+^ supplementation enhances β‐cell proliferation but not β‐cell size in TRPM7 kinase‐dead mice. (A) Pancreas weight of WT and *Trpm7* ^*R/R*^ mice after 8 weeks of HFD and 8 weeks of different dosages of supplementary Mg^2+^ (*n* ≥ 3 mice per genotype). (B) Number of islets per pancreatic cryosection (*n* ≥ 60 slides, at least three mice per genotype) (C) β‐cell size ( ≥ 10 islets, at least five mice per genotype) and (D) percentage of Ki67‐positive cells from the population (100%) of the insulin‐positive cells per pancreatic islet in WT and *Trpm7* ^*R/R*^ mice after 8 weeks of HFD and 8 weeks of different dosages of supplementary Mg^2+^ (*n* = 20, four mice per genotype). (E) Confocal images of WT and *Trpm7* ^*R/R*^ islets stained for DAPI (blue), insulin (green), and Ki67 (red). The scale bar represents 100 μm. Data show means ± SEM and statistical differences were assessed by one‐way ANOVA. Circles in bar graphs represent single values. *p* values are shown above the bars.

Next, we asked whether Mg^2+^ supplementation could improve the impaired compensatory pathways in *Trpm7* ^*R/R*^ mice. We assessed the impact of Mg^2+^ supplementation on the number of islets per pancreatic section, β‐cell mass, and cell proliferation. Although total pancreatic weight did not differ between high‐fat diet–fed *Trpm7* ^*R/R*^ mice and their control littermates receiving either the 0.22% or 0.75% Mg^2+^ diet (Figure 4A), a significant reduction in the number of islets per pancreatic section and β‐cell mass was detected in *Trpm7* ^*R/R*^ mice compared to their control littermates under both dietary conditions (Figure 4B,C). To assess β‐cell proliferation, Ki67 was used as a marker. The abundance of Ki67‐positive β‐cells was markedly reduced in *Trpm7* ^*R/R*^ islets compared to control littermates when animals were on 0.22% Mg^2+^ diet (Figure 4C,D). These findings suggest that the loss of TRPM7 kinase activity restricts β‐cell proliferation in response to an HFD. However, when animals received Mg^2+^ supplementation, no significant difference in β‐cell proliferation was observed between *Trpm7* ^*R/R*^ islets and those of control littermates. Notably, *Trpm7* ^*R/R*^ mice showed a significant improvement in β‐cell proliferation after receiving 0.75% Mg^2+^ compared to *Trpm7* ^*R/R*^ mice on the standard dietary Mg^2+^ (Figure 4A).

### 3.5 Pharmacological Inhibition of TRPM7 Impairs β‐Cell Proliferation via Reduced PDX1 Expression

**Figure 5:**
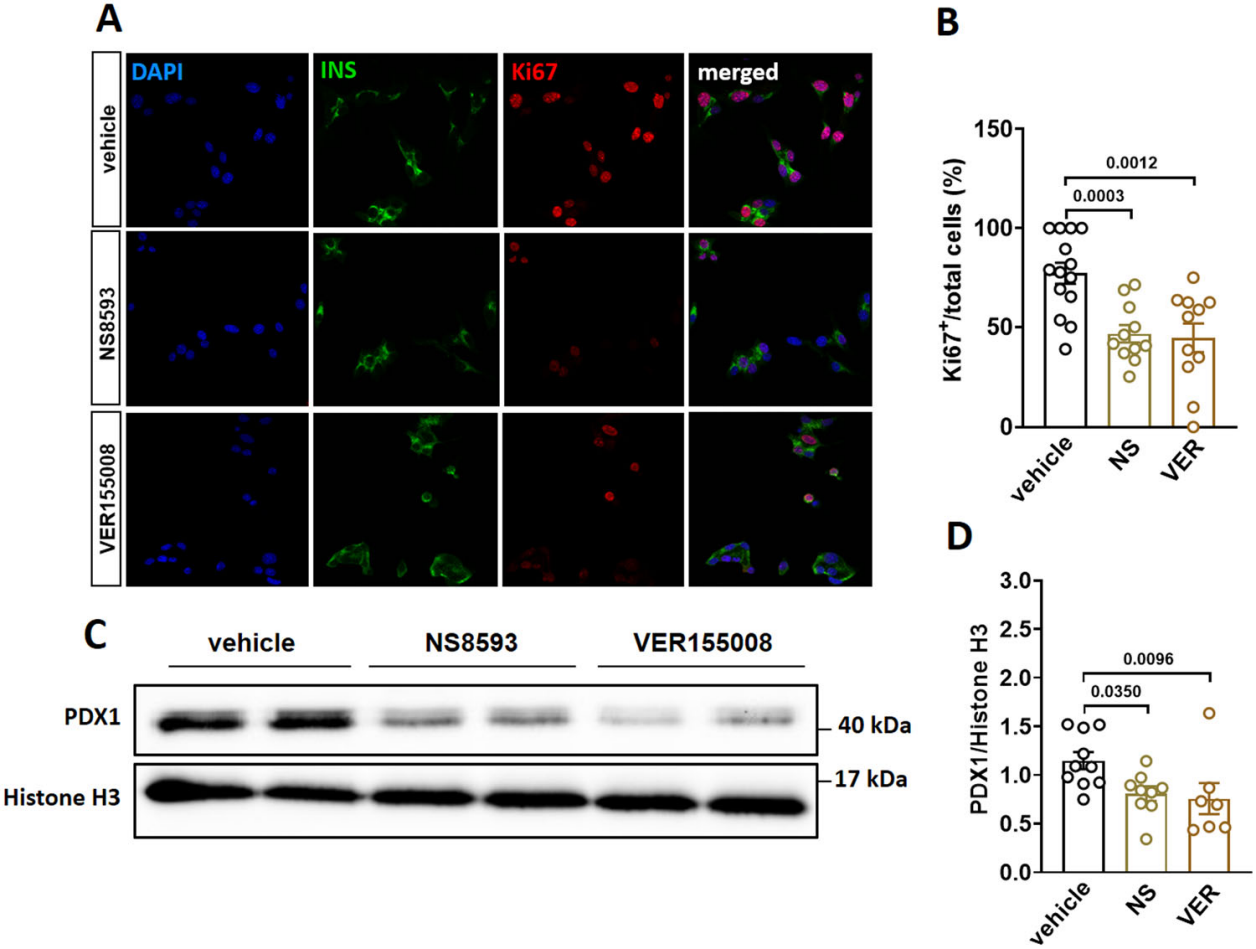
Pharmacological inhibition of TRPM7 impairs β‐cell proliferation and reduces protein expression of PDX1 in MIN6 cells. (A) Confocal images of MIN6 cells after incubation with DMSO (vehicle) and 30 µM NS8593 and 10 µM VER155008, stained for DAPI (blue), insulin (green), and Ki67 (red). (B) Percentage of Ki67‐positive cells from the population (100%) of the insulin‐positive cells (at least 12 slides per condition). (C) Lysates from MIN6 cells that had been treated with DMSO (vehicle) and 30 µM NS8593 and 10 µM VER155008 (TRPM7 antagonists) were subjected to western blot analysis. Blots were probed with the indicated antibodies. Histone H3 was used as a loading control. (D) Quantification of the western blot analysis data shown in (C). Data show means ± SEM and statistical differences were assessed by one‐way ANOVA. Circles in bar graphs represent single values. *p* values are shown above the bars.

In light of our in vivo findings of diminished β‐cell proliferation in islets isolated from *Trpm7* ^*R/R*^ mice, we explored this effect in vitro using MIN6 cells and two well‐established TRPM7 blockers, NS8593 (Chubanov and Gudermann 2020) and VER155008 (Rössig et al. 2022). Pre‐incubation of MIN6 cells with either 30 µM NS8593 or 10 µM VER155008 for 24 h resulted in a 39% and 42% reduction, respectively, in Ki67‐positive MIN6 cells (Figure 5A,B). Due to the critical role of PDX1 in cell proliferation in pancreatic β‐cells, we investigated whether TRPM7 inhibition affects this protein expression level in MIN6 cells. Western blot analysis revealed that treatment with either 30 µM NS8593 or 10 µM VER155008 significantly reduced PDX1 expression in MIN6 cells (Figure 5C,D).

### 3.6 Mg^2+^ Supplementation Restores Reduced ERK1/2 Phosphorylation Caused by TRPM7 Inhibition

**Figure 6:**
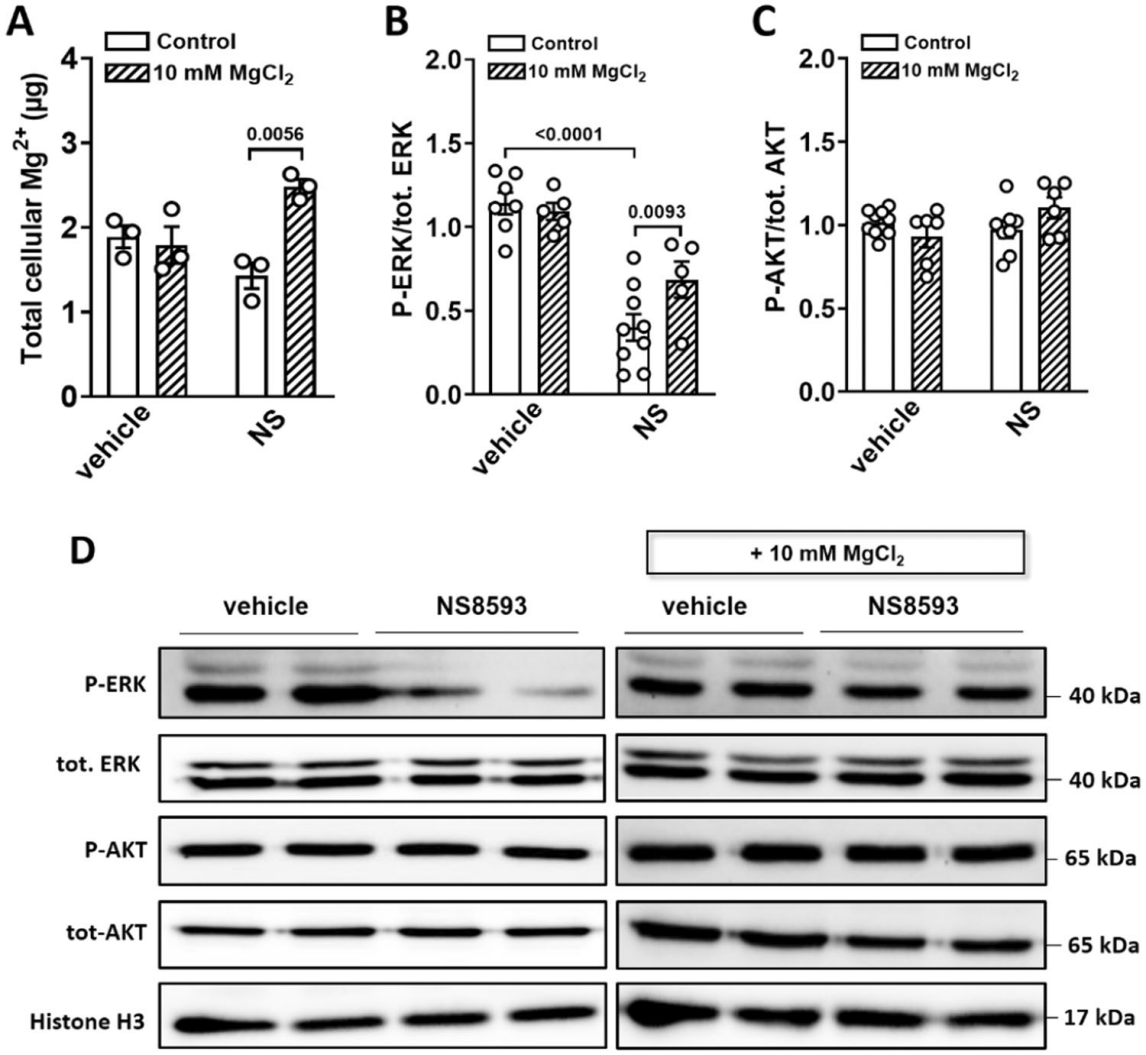
Mg^2+^ supplementation restores impaired ERK1/2 phosphorylation caused by the pharmacological inhibition of TRPM7. (A) Cellular Mg^2+^ content was measured by ICP‐MS. MIN6 cells treated with DMSO (vehicle) or 30 μM NS8593 (NS) were cultured in regular media with or without supplementation with 10 mM MgCl_2_ for 24 h ahead of sampling (*n* = 3). Data represent the total cellular Mg^2+^ (µg) in 5 million cells. (B−D) Western blot analysis was performed on lysates from MIN6 cells treated with DMSO or 30 µM NS8593, with or without 10 mM MgCl_2_ (*n* ≥ 5). Blots were probed with the indicated antibodies. Histone H3 was used as a loading control. Data show means ± SEM and statistical differences were assessed by one‐way ANOVA. Circles in bar graphs represent single values. *p* values are shown above the bars.

Since AKT and ERK1/2 are suggested to be downstream signaling targets of TRPM7, we assessed the phosphorylation levels of these proteins in MIN6 cells in the presence of NS8593. Western blot analysis revealed that treatment with 30 µM NS8593 significantly reduced ERK1/2 phosphorylation in MIN6 cells, while AKT phosphorylation remained unaffected by this blocker (Figure 6A−C). Next, we increased the MgCl_2_ concentration in the medium from 1 mM to 10 mM and incubated the cells with 30 µM NS8593 for 24 h. First, we assessed the intracellular Mg^2+^ concentration in MIN6 cells after 24 h of exposure to the TRPM7 inhibitor, with or without Mg^2+^ supplementation. Although treatment with 30 µM NS8593 reduced intracellular Mg^2+^ levels relative to the vehicle control, this difference did not reach statistical significance. However, Mg^2+^ supplementation effectively restored intracellular Mg^2+^ levels in cells treated with NS8593 (Figure 6A). Notably, the increased Mg^2+^ concentration improved ERK1/2 phosphorylation in MIN6 cells (Figure 6B−D). These results indicate that increased Mg^2+^ supplementation partially reversed the phosphorylation decline caused by pharmacological blockade of TRPM7.

## 4 Discussion

TRPM7 is the first molecularly defined component of the mammalian Mg^2+^ transport system and is regulated by the dynamic interaction between its channel and kinase domains (Schmitz et al. 2003). The channel moiety of TRPM7 is a primary pathway for cellular Mg^2+^ uptake, while fluctuations in Mg^2+^ concentration near the channel pore can modulate kinase domain activity (Ryazanova et al. 2004; Schmitz et al. 2003). Furthermore, the TRPM7 kinase domain is essential for regulation of channel activity, stability, and cellular trafficking (Cai et al. 2018; Demeuse et al. 2006). Accumulating evidence suggests that Mg^2+^ supplementation alleviates impaired phenotypes caused by TRPM7 disruption (Gupta et al. 2023; Ryazanova et al. 2010; Schmitz et al. 2003; Yee et al. 2011). In this study, we investigated whether Mg^2+^ supplementation could mitigate the impaired metabolic phenotype linked to TRPM7 kinase deficiency. Our results revealed that *Trpm7* ^*R/R*^ mice fed an obesogenic diet with standard Mg^2+^ levels exhibit glucose intolerance compared to their control littermates. In contrast, increasing Mg^2+^ intake improved glucose tolerance in *Trpm7* ^*R/R*^ mice on an obesogenic diet. Notably, Mg^2+^ supplementation had no effect on glucose tolerance in WT mice.

Type 2 diabetes is characterized by a combination of peripheral insulin resistance and reduced secretion of insulin. Our recent study demonstrated that the impaired glucose tolerance observed in *Trpm7* ^*R/R*^ mice is partially attributable to reduced insulin production and secretion (Khajavi, Beck, et al. 2023). We initially hypothesized that the improved glucose tolerance in *Trpm7* ^*R/R*^ mice treated with supplementary Mg^2+^ results from its beneficial effects on insulin secretion. However, our ex vivo analysis revealed that Mg^2+^ supplementation did not significantly improve glucose‐ or tolbutamide‐induced insulin secretion in islets isolated from *Trpm7* ^*R/R*^ mice compared to their control littermates. This negative finding is consistent with previous studies demonstrating that acute and chronic Mg^2+^ deficiency does not impair GIIS (Gommers et al. 2019; Khajavi, Riçku, et al. 2023). Our research, along with studies from others, has shown that acute lowering of extracellular Mg^2+^ concentrations does not affect GIIS in islets isolated from C57BL/6 mice or in insulin‐secreting INS‐1 cells (Gommers et al. 2019; Khajavi, Riçku, et al. 2023). In addition, GIIS remained unchanged in isolated islets from *Trpm6* ^*Δ17/fl*^ *;Villin1‐Cre* mice, a well‐established mouse model of hypomagnesemia, indicating that chronic Mg^2^ deficiency does not impair the secretory function of pancreatic β‐cells (Khajavi, Riçku, et al. 2023). Thus, we propose here that the reduced insulin secretion in TRPM7 kinase‐deficient mice is not due to impaired Mg^2+^ uptake via the channel moiety of TRPM7. This observation may explain why Mg^2+^ supplementation fails to improve GIIS in *Trpm7* ^*R/R*^ mice. Therefore, we hypothesize that the beneficial effects of Mg^2^ are independent of the insulin secretion machinery and may instead involve alternative mechanisms.

Adequate intracellular Mg^2+^ levels are essential for the phosphorylation of insulin receptors, whereas dysregulated Mg^2+^ balance disrupts insulin receptor signaling, and ultimately reduces peripheral insulin sensitivity (Takaya et al. 2004). Numerous studies demonstrated that elevated intracellular Mg^2+^ concentrations enhance insulin receptor tyrosine kinase (RTK) activity and improve glucose transport in target cells (Cutler et al. 2019; Siddiqui et al. 2016). Activation of the insulin receptor triggers a wide range of downstream signaling pathways, including the PI3K/AKT and Raf/MEK/ERK1‐2 (De Meyts 2000). In adipocytes, Mg^2+^ plays a pivotal role in insulin‐mediated glucose uptake by enhancing AKT activation and promoting the translocation of GLUT4 to the plasma membrane (Oost et al. 2022). In rat skeletal muscle, Mg^2+^ supplementation increases the expression levels of GLUT4 (Morakinyo et al. 2018). TRPM7 and its homolog TRPM6 are closely associated with RTK‐mediated signaling pathways. A growing body of evidence highlights TRPM7 as a key regulator of RTK downstream signaling, functioning through both its channel and kinase domains (Zou et al. 2019).

Recently, we reported that *Trpm7*‐kinase‐deficient mice exhibit hyperglycemia and impaired plasma insulin levels (Khajavi, Beck, et al. 2023). Here, we hypothesized that the improved glucose tolerance observed with Mg^2+^ supplementation results from its beneficial effects on insulin receptor signaling and glucose transport in target cells, thereby alleviating the dysfunctional metabolic phenotype of *Trpm7* ^*R/R*^ mice. To test this hypothesis, we examined the effects of two different dietary Mg^2+^ concentrations on the metabolic phenotype of *Trpm7* ^*R/R*^ mice. Supplementary Mg^2+^ remarkably reduced fasting blood glucose in *Trpm7* ^*R/R*^ mice relative to the *Trpm7* ^*R/R*^ mice under the standard Mg^2+^ diet. Notably, fasting blood glucose levels in WT mice remained unchanged regardless of the dietary Mg^2+^ concentration. In addition, supplementary Mg^2+^ eliminated the differences in both fasting and fed blood glucose levels, as well as plasma insulin levels that were previously observed between *Trpm7* ^*R/R*^ and WT mice (Khajavi, Beck, et al. 2023). Reductions in fasting blood glucose and plasma insulin levels are indicative of enhanced insulin sensitivity (Ahrén. 2007). Thus, we propose that elevated serum Mg^2+^ levels enhance insulin sensitivity, thereby contributing to improved glycemic control in high‐fat diet‐fed *Trpm7* ^*R/R*^ mice. This observation is also in line with a recent meta‐analysis that failed to show any significant effect of Mg^2+^ supplementation compared to placebo in improving insulin secretion, while the main action of Mg^2+^ appears to be attributable to improved insulin sensitivity and HOMA‐IR, particularly in individuals at high risk of diabetes (Veronese et al. 2021). Nevertheless, the precise roles of the TRPM7 channel and its kinase domain in this process remain to be elucidated, as their contributions may involve complex regulatory mechanisms in target cells.

Previous research has shown that extracellular Mg^2+^ supplementation can effectively mitigate proliferation arrests in *Trpm7*‐kinase deficient cells (Ryazanova et al. 2010). This prompted us to investigate whether improved glucose metabolism in *Trpm7* ^*R/R*^ mice is attributable to increased β‐cell proliferation. While earlier research has shown that TRPM7 kinase‐deficient cells can allow sufficient Mg^2+^ entry to maintain total cellular Mg^2+^ levels and support normal cell division (Schmitz et al. 2003), our immunostaining analysis revealed that Mg^2+^ supplementation enhances β‐cell proliferation in *Trpm7* ^*R/R*^ islets. We propose that this discrepancy may stem from the fact that their findings were predominantly derived from experiments using the DT‐40 cell line, which may not represent the complexity of pancreatic β‐cells. Here, we suggest that elevated serum Mg^2+^ levels promote β‐cell proliferation, contributing to improved glycemic control in *Trpm7* ^*R/R*^ mice fed an HFD.

To validate our hypothesis in vitro, we employed a pharmacological approach with MIN6 cells. Our analysis revealed that the application of TRPM7 blockers, NS8593 and VER155008, led to a decrease in β‐cell proliferation. NS8593 and VER155008 are potent small‐molecule inhibitors known to significantly reduce TRPM7‐mediated current amplitudes. Both inhibitors bind to a shared vanilloid‐like site on TRPM7, and their mechanism of action involves stabilizing the channel's closed state, as indicated by the similarity between the inhibitor‐bound and resting states (Nadezhdin et al. 2023). While VER155008 has recently been identified as a TRPM7 inhibitor (Rössig et al. 2022), studies examining its effects on TRPM7 kinase function are still limited. Conversely, extensive research on NS8593 has demonstrated that TRPM7 channel activity is integral to its kinase function. For instance, studies on HEK293 cells overexpressing hTRPM7 revealed that NS8593 treatment resulted in a 42% reduction in hTRPM7 autophosphorylation. This finding highlights the interplay between TRPM7 channel activity and its kinase function, showing that NS8593 not only blocks ion flow through the channel but also significantly reduces kinase activity (Faouzi et al. 2017). Additionally, recent research has shown that NS8593‐mediated TRPM7 blockade inhibits RhoA activation via the TRPM7 kinase domain. This inhibition leads to reduced transcriptional activity of Myocardin‐related transcription factor A (MRTF‐A), a crucial regulator of genes involved in cell proliferation (Franz et al. 2024). In this study, we suggest that inhibition of the TRPM7 channel domain via both blockers significantly inhibits the kinase function leading to the reduced *Pdx1* expression in pancreatic β‐cells. Previous studies have linked the overexpression of PDX1 to the upregulation of several cell cycle genes leading to increased β‐cell proliferation (Hayes et al. 2013). PDX1 phosphorylation is known to be triggered by PI3K/AKT and ERK1/2 signaling (Jara et al. 2020; Khoo et al. 2003), and TRPM7 kinase is recognized as a key regulator of these pathways (Nadolni et al. 2020; Zhang et al. 2017). Here, we found that inhibiting TRPM7 in MIN6 cells resulted in a reduction in PDX1 expression and disruption of ERK1/2 signaling pathways. This observation is in line with our previous findings that the kinase moiety of TRPM7 regulates the TGF‐β/SMAD signaling pathway activity and *Pdx1* expression in islets isolated from *Trpm7* ^*R/R*^ and β*Trpm7* KO mice (Khajavi, Beck, et al. 2023).

A recent study demonstrated that treatment with NS8593 in human T lymphocytes leads to a decrease in intracellular Mg^2+^ levels, which could be restored through Mg^2+^ supplementation (Hoelting et al. 2024). Similarly, our findings revealed a decline in intracellular Mg^2+^ levels, which could be effectively reversed with Mg^2+^ supplementation. Strikingly, Mg^2+^ supplementation reversed the reduced ERK1/2 phosphorylation observed in MIN6 cells treated with NS8593. Therefore, we suggest that elevating extracellular Mg^2+^ levels can partially mitigate the transcriptional and signaling impairments caused by pharmacological inhibition of TRPM7. Notably, previous studies reported that elevated Mg^2^ levels can enhance ERK/CREB signaling in other cell types, such as neural progenitor cells (Liao et al. 2017). Therefore, while our in vitro findings suggest that Mg^2+^ supplementation restores TRPM7 kinase functionality and promotes ERK1/2 phosphorylation, these results do not entirely exclude the possibility that increased Mg^2+^ availability may induce ERK1/2 phosphorylation through TRPM7‐independent mechanisms.

In conclusion, our findings demonstrate that Mg^2+^ supplementation enhances glucose metabolism and promotes β‐cell proliferation in TRPM7 kinase‐deficient mice. This effect may be driven by two potential mechanisms: (1) the loss of TRPM7 kinase function in *Trpm7* ^*R/R*^ mice may impair Mg^2+^ uptake, and Mg^2+^ supplementation partially rescues this phenotype by restoring cellular Mg^2+^ homeostasis; or (2) increased Mg^2+^ availability may enhance compensatory signaling pathways that mitigate the metabolic dysfunction associated with TRPM7 kinase deficiency. Elucidating the precise molecular mechanisms underlying these effects remains a key objective for future investigations. Importantly, our pharmacological analysis in MIN6 cells revealed that elevated extracellular Mg^2+^ concentrations restore impaired signaling pathways caused by TRPM7 disruptions. This underscores the potential of Mg^2+^ to reverse the adverse metabolic and cellular phenotypes associated with the loss of TRPM7 function.

## Author Contributions

Noushafarin Khajavi and Thomas Gudermann contributed to the conception and design of the study. Noushafarin Khajavi, Severin Boulassel, Pascale C. F. Schreier, Anna M. Melyshi, Katharina Jacob, and Johanna Berger performed and analyzed the experiments. Noushafarin Khajavi wrote the first draft of the manuscript. Thomas Gudermann, Peter S. Reinach, Andreas Breit, Susanna Zierler, Ingrid Boekhoff, and Timo D. Müller critically revised and edited the final version of the manuscript. All authors have read and approved the final version of the manuscript and agree to be accountable for all aspects of the work in ensuring that questions related to the accuracy or integrity of any part of the work are appropriately investigated and resolved. All persons designated as authors qualify for authorship, and all persons who qualify for authorship are listed.

## Ethics Statement

All animal experiments were performed in accordance with the EU Animal Welfare Act and were approved by the District Government of Upper Bavaria, Munich, Germany, on animal care (permit no. 55.2‐2532.Vet_02‐19‐035).

## Conflicts of Interest

T.D.M. receives research funding from Novo Nordisk; however, these funds are unrelated to the work described here. T.D.M. further received speaking fees within the past 3 years from Novo Nordisk, Eli Lilly, AstraZeneca, Merck, Berlin Chemie AG, and Mercodia. The other authors declare no conflicts of interest.

## Data Availability

All data will be shared by the corresponding author upon reasonable request.
